# Knowledge, attitudes, and practices regarding floaters among patients

**DOI:** 10.3389/fmed.2025.1579435

**Published:** 2025-07-09

**Authors:** Bin Zhang, Hangyu Liu, Haijing Zhu, Xianyong Sun, Rongyu Gao

**Affiliations:** ^1^Weifang Eye Hospital, National Key Clinical Specialty, Zhengda Guangming Eye Group, Weifang, China; ^2^Weifang Eye Institute, Weifang, China; ^3^Laizhou Zhengda Guangming Eye Hospital, Zhengda Guangming Eye Group, Laizhou, China; ^4^Weifang Zhengda Guangming Eye Hospital, Zhengda Guangming Eye Group, Weifang, China

**Keywords:** knowledge, attitude and practice, questionnaires, floaters, myodesopsia, cross-sectional study

## Abstract

**Objectives:**

This study aimed to investigate the knowledge, attitudes, and practices (KAP) regarding floaters among patients with floaters.

**Methods:**

This cross-sectional study enrolled participants diagnosed with floaters between April 2023 and October 2023 in Weifang, China. Demographic information and KAP regarding floaters were collected via web-based questionnaires.

**Results:**

A total of 430 respondents were included, with 251 (58.37%) females. Of the respondents, 270 (62.79%) experienced their first episode of floaters for less than 1 year. Mean knowledge, attitude, and practice scores were 8.81 ± 4.20 (possible range: 0–13), 17.23 ± 5.05 (possible range: 6–30), and 14.67 ± 2.95 (possible range: 4–20), respectively. Correlation analyses revealed a significant positive relationship between knowledge and practices (*r* = 0.239, *p* < 0.001), whereas attitudes were inversely associated with practices (*r* = −0.219, *p* < 0.001). The structural equation model revealed that knowledge had a direct positive influence on practices (*β* = 0.403, *p* < 0.001), while attitudes exhibited a direct negative impact on practices (*β* = −0.112, *p* < 0.001).

**Conclusion:**

Patients demonstrated suboptimal knowledge and negative attitudes, but many engaged in proactive practices to manage floaters. Interventions focusing on enhancing knowledge and fostering positive attitudes among individuals with floaters are recommended to improve clinical practices.

## Introduction

In the broader landscape of ocular health, the phenomenon of floaters, characterized by the perception of dark shadows or spots drifting across the field of vision, stands as a prevalent yet frequently overlooked concern (1). While vitreous floaters are common, they usually cause minimal discomfort for the majority of individuals (2). However, a notable subset experiences distressing symptoms. This becomes evident in the aging population, where vitreous floaters become more frequent due to degenerative vitreous changes throughout life (1, 2). Ocular floaters’ prevalence ranges from 27 to 63%, exerting a considerable impact on individuals’ overall wellbeing (3). A study revealed a 76% prevalence of vitreous floaters in the general population (4). Notably, while many patients report mild or no distress, 76% experience anxiety related to floaters, and 33% report a significant decrease in their overall quality of life (5). This underscores patients’ perception that floaters represent a substantial medical condition with a profound negative influence on both vision and overall quality of life (6).

Despite their impact on daily life and visual wellbeing, a significant gap persists in our understanding of the knowledge, attitudes, and practices (KAP) surrounding floaters. The structured survey method of KAP offers a valuable framework for delving into the intricacies of how individuals perceive and manage floaters (7, 8). Remarkably, within the existing body of literature, there is a noticeable dearth of KAP studies specifically focused on floaters, highlighting an unexplored domain in ophthalmic research. Furthermore, gaining insights into patients’ knowledge, attitudes, and practices concerning floaters can provide healthcare professionals with essential information regarding patient needs and expectations. This is crucial for formulating more effective clinical management and communication strategies.

Therefore, this study aims to investigate the KAP regarding floaters among patients with this condition.

## Materials and methods

### Study design and participants

This cross-sectional study was conducted between 25 January and 17 April 2024, at Weifang Eye Hospital of Sun Optical Vision Group, Laizhou Sun Optical Vision Hospital, and Weifang Weicheng Sun Optical Vision Hospital. Participants diagnosed with floaters were included in the study. The inclusion criteria consisted of individuals aged 18 years and above, experiencing symptoms of floating dark shadows in one or both eyes, and demonstrating normal mental and intellectual conditions. Exclusion criteria applied to participants with questionnaire completion times of less than 90 s and those with a history of other intraocular surgeries. To ensure accurate screening, participants’ medical records were reviewed prior to enrollment to identify any documented mental or intellectual disorders and previous intraocular surgeries. In addition, questionnaire completion time was automatically recorded by the online system, and responses submitted in less than 90 s were excluded during data cleaning to ensure data quality and response authenticity.

This study was approved by the Medical Ethics Committee of Zhengda Guangming Ophthalmology Group (2023-01-03), and all participants provided written informed consent.

### Questionnaire

The questionnaire was designed based on the published literature (9–11). Following the creation of the initial draft, a pilot test was conducted on a limited scale with 50 participants to assess its reliability. The reliability analysis yielded Cronbach’s *α* coefficients of 0.838, 0.617, and 0.757 for the knowledge, attitude, and practice sections, respectively, with an overall coefficient of 0.781, indicating acceptable internal consistency. The questionnaire was originally designed in Chinese and administered in Chinese to all participants, as this is the native language of the study population. The content was later translated into English solely for publication and to facilitate understanding for international readers.

The final questionnaire, presented in Chinese, comprised four dimensions for information collection, encompassing a total of 39 items. These dimensions included 14 items for gathering basic information, 14 items for assessing knowledge, 6 items for exploring attitudes, and 5 items for evaluating practices. During statistical analysis, scores were assigned based on the response options for each item. Specifically, within the knowledge dimension, correct responses received a score of 1, while incorrect or unclear responses were assigned a score of 0. Notably, item 14 served as a trap question designed to identify illogical responses and was subsequently excluded from the knowledge dimension score, resulting in a score range of 0 to 13 points. The attitude dimension was assessed using a 5-point Likert scale, with scores spanning from very positive (5 points) to very negative (1 point), yielding a total possible score range of 6 to 30 points. Similarly, the practice dimension also used a 5-point Likert scale, with scores ranging from 4 to 20; however, it is important to note that the first item was included for descriptive purposes only. Attaining scores exceeding 70% of the maximum in each section signified a commendable level of knowledge, positive attitudes, and proactive practices (12). Construct validity was assessed using confirmatory factor analysis (CFA). The model demonstrated acceptable fit (CMIN/DF = 3.586; RMSEA = 0.078; IFI = 0.874; TLI = 0.859; CFI = 0.873), indicating good structural validity. Factor loadings of items under each dimension were all statistically significant (*p* < 0.001) except for one item in the practice dimension (P5, *p* = 0.132), which was retained for its clinical relevance (Supplementary Table S1). Additionally, the Kaiser–Meyer–Olkin (KMO) value was 0.891 (*p* < 0.001), supporting the adequacy of the data for factor analysis.

### Questionnaire distribution

The questionnaire was distributed using an online survey platform, Questionnaire Star, in China. Participants accessed and completed the electronic questionnaire by scanning a QR code. To ensure reliable and complete results, each IP address was restricted to one submission, and all items required responses.

### Sample size calculation

The sample size was determined using the standard formula for cross-sectional studies: *n = Z^2^P(1 − P)/d^2^*, where *Z* is the standard normal variate (1.96 for 5% type I error), *P* is the estimated prevalence of floaters in the general population (approximately 45%), and *d* is the absolute precision (5%). This yielded a minimum required sample size of 384 participants. After accounting for a 10% non-response rate, the adjusted sample size was 427 participants. Ultimately, 430 valid responses were obtained, exceeding the calculated minimum and ensuring sufficient statistical power for analysis.

### Statistical analysis

Statistical analysis was performed using SPSS 26.0 (IBM Corp., Armonk, NY, USA). Continuous variables were expressed as mean ± standard deviation (SD), and between-group comparisons were conducted using *t*-tests or analysis of variance (ANOVA). Categorical variables were presented as *n* (%). Spearman’s correlation analysis was used to evaluate correlations between knowledge, attitude, and practice scores. Univariate and multivariate logistic regression analyses were utilized to explore risk factors associated with distinct components of knowledge (K), attitudes (A), and practices (P), with 70% of KAP scores as the cutoff. Additionally, a structural equation model (SEM) was used to scrutinize the interrelations among the questionnaire dimensions, operating under the assumption that knowledge exerts a direct influence on attitudes and practices, while attitudes directly impact practices. Two-sided *p*-values of <0.05 were considered statistically significant in this study.

## Results

### Basic characteristics

In this study, data were gathered from 430 questionnaires, of which 251 (58.37%) respondents were female, 350 (81.40%) held stable jobs, 130 (30.23%) were not myopic, and 270 (62.79%) had experienced their first episode of floaters for less than 1 year. The mean knowledge, attitude, and practice scores were 8.81 ± 4.20 (possible range: 0–13), 17.23 ± 5.05 (possible range: 6–30), and 14.67 ± 2.95 (possible range: 4–20), respectively. Demographic analyses showed that age, education, employment status, and duration since first experiencing floaters significantly influenced knowledge, attitudes, and practice scores. Disparities in residence, monthly per capita income, and smoking status were found to impact knowledge scores. Moreover, variations in gender, marital status, residence, monthly per capita income, myopia status, alcohol consumption, and blood sugar abnormality were associated with different attitudes. Additionally, differences in gender, myopia status, smoking, and alcohol consumption influenced distinct practices (all *p*-values less than 0.05) (Table 1).

**Table 1:** Baseline and KAP scores.

Variables	*N* (%)	Knowledge, mean ± SD	*p*	Attitude, mean ± SD	*p*	Practice, mean ± SD	*p*
Total score (*n* = 430)		8.81 ± 4.20		17.23 ± 5.05		14.67 ± 2.95	
Age			0.031		<0.001		0.002
18–40 years	193 (44.88)	9.28 ± 3.99		18.71 ± 5.52		14.40 ± 3.05	
41–60 years	211 (49.07)	8.28 ± 4.35		16.22 ± 4.34		14.68 ± 2.88	
Over 60 years	26 (6.05)	9.69 ± 4.02		14.50 ± 3.58		16.54 ± 2.10	
Sex			0.267		0.010		0.043
Male	179 (41.63)	8.55 ± 4.50		17.68 ± 5.09		14.32 ± 2.90	
Female	251 (58.37)	9.00 ± 3.96		16.70 ± 4.97		14.91 ± 2.97	
Marital status			0.133		0.016		0.655
Single	57 (13.26)	9.16 ± 4.24		18.96 ± 5.15		14.77 ± 2.80	
Married	361 (83.95)	8.84 ± 4.16		17.00 ± 5.03		14.67 ± 2.97	
Divorced or widowed	12 (2.79)	6.50 ± 4.80		16.00 ± 3.77		13.92 ± 3.18	
Residence			0.001		0.001		0.709
Rural	82 (19.07)	7.29 ± 4.86		15.46 ± 3.99		14.78 ± 2.88	
Urban	327 (76.05)	9.13 ± 3.97		17.70 ± 5.26		14.61 ± 2.96	
Suburban	21 (4.88)	9.90 ± 3.52		16.86 ± 4.05		15.10 ± 3.21	
Education			<0.001		<0.001		0.004
Primary school and below	18 (4.19)	7.72 ± 4.56		13.67 ± 3.03		15.22 ± 2.60	
Junior high school	51 (11.86)	7.16 ± 4.98		14.33 ± 4.10		15.94 ± 2.59	
High school/Technical school	59 (13.72)	6.92 ± 4.83		16.36 ± 3.81		15.05 ± 2.74	
College/Bachelor’s degree	256 (59.53)	9.15 ± 3.84		17.32 ± 4.83		14.30 ± 3.07	
Master’s degree and above	46 (10.70)	11.63 ± 1.37		22.48 ± 5.15		14.59 ± 2.68	
Employment status			0.001		<0.001		0.003
Employed with stable job	350 (81.40)	9.13 ± 3.95		17.65 ± 5.10		14.47 ± 2.97	
Unemployed or irregular employment	80 (18.60)	7.41 ± 4.93		15.41 ± 4.43		15.54 ± 2.73	
Monthly *Per Capita* income, RMB			0.013		<0.001		0.335
<2000	37 (8.60)	7.84 ± 4.48		16.14 ± 4.17		14.14 ± 3.41	
2,000–5,000	141 (32.79)	8.16 ± 4.23		15.95 ± 4.32		14.79 ± 2.93	
5,000–10,000	161 (37.44)	8.95 ± 4.20		17.48 ± 5.03		14.78 ± 2.88	
10,000–20,000	61 (14.19)	10.02 ± 3.89		18.64 ± 5.88		14.13 ± 2.80	
>20,000	30 (6.98)	9.90 ± 3.60		20.43 ± 5.52		15.17 ± 3.14	
Myopia status			0.142		0.043		0.013
Not myopic	130 (30.23)	8.19 ± 4.57		16.08 ± 4.56		15.41 ± 2.87	
Myopia with a degree of less than 100	28 (6.51)	8.54 ± 3.95		18.18 ± 5.35		14.89 ± 2.66	
Myopia with a degree of 100–299	88 (20.47)	8.86 ± 4.20		17.77 ± 5.30		14.47 ± 2.96	
Myopia with a degree of 300–499	92 (21.40)	8.73 ± 4.13		17.34 ± 4.74		14.13 ± 2.90	
Myopia with a degree of 500–799	68 (15.81)	9.81 ± 3.69		18.24 ± 5.42		14.10 ± 3.14	
Myopia with a degree of ≥800	24 (5.58)	9.83 ± 3.61		17.13 ± 5.75		14.75 ± 2.74	
Smoking status			<0.001		0.445		0.002
Never smoked	349 (81.16)	9.15 ± 4.02		17.10 ± 5.06		14.91 ± 2.84	
Former smoker, currently quit	30 (6.98)	8.43 ± 4.58		17.37 ± 4.83		13.70 ± 3.32	
Currently still smoking	51 (11.86)	6.73 ± 4.57		18.06 ± 5.16		13.59 ± 3.16	
Alcohol consumption			0.080		0.003		0.010
Frequent alcohol consumption	40 (9.30)	7.40 ± 4.87		17.93 ± 4.81		14.10 ± 3.11	
Occasional alcohol consumption	122 (28.37)	8.90 ± 4.22		18.42 ± 5.23		14.11 ± 2.66	
Rarely or never drinks	268 (62.33)	8.99 ± 4.05		16.59 ± 4.91		15.00 ± 3.01	
Blood sugar abnormality			0.285		0.029		0.848
Insulin resistance	10 (2.33)	9.20 ± 3.33		15.60 ± 5.04		15.00 ± 2.31	
Prediabetes	12 (2.79)	9.42 ± 4.72		14.92 ± 4.89		15.33 ± 2.23	
Diabetes	26 (6.05)	7.31 ± 4.81		15.15 ± 3.84		14.54 ± 2.85	
None	382 (88.84)	8.89 ± 4.15		17.49 ± 5.09		14.64 ± 3.00	
Diagnosed hypertension			0.291		0.504		0.254
Yes	46 (10.70)	8.20 ± 4.92		16.76 ± 4.88		14.20 ± 3.41	
No	384 (89.30)	8.89 ± 4.10		17.29 ± 5.08		14.72 ± 2.89	
History of eye trauma			0.559		0.091		0.977
Yes	19 (4.42)	8.26 ± 4.72		15.32 ± 4.60		14.68 ± 2.93	
No	411 (95.58)	8.84 ± 4.18		17.32 ± 5.06		14.66 ± 2.96	
Duration since first experiencing floaters			0.034		<0.001		0.003
Less than 1 year	270 (62.79)	8.41 ± 4.43		16.02 ± 4.47		15.00 ± 2.80	
1–2 years	46 (10.70)	8.85 ± 3.81		18.93 ± 5.25		13.33 ± 2.88	
2–3 years	26 (6.05)	10.04 ± 3.18		19.15 ± 5.21		13.81 ± 3.31	
3–5 years	28 (6.51)	10.57 ± 2.90		19.79 ± 5.54		14.86 ± 3.59	
More than 5 years	60 (13.95)	9.27 ± 4.05		19.35 ± 5.47		14.45 ± 2.92	

### Knowledge, attitudes, and practices

The distribution of knowledge dimensions revealed that the question with the highest correctness rate was “Patients with high myopia and floaters should regularly undergo fundus examinations at the hospital” (K10), with 79.07%. Conversely, the question with the lowest correctness rate was “Floaters can cause discomfort symptoms such as dizziness” (K5), with 47.21% (Table 2).

**Table 2:** Responses to knowledge dimension items.

Items, *n* (%)	Correct rate
1. Floaters are generally caused by vitreous degeneration, which is an aging phenomenon.	285 (66.28)
2. Vitreous liquefaction and posterior vitreous detachment are the main causes of floaters.	254 (59.07)
3. Floaters can be transmitted to people around you.	326 (75.81)
4. Floaters may be limited to one eye or may occur in both eyes.	331 (76.98)
5. Floaters can cause discomfort symptoms such as dizziness.	203 (47.21)
6. Floaters often occur in middle-aged and elderly people over 40, highly myopic individuals, and those who have had cataract surgery.	257 (59.77)
7. Conditions such as high blood pressure, diabetes, intraocular inflammation, retinal holes, and trauma can also lead to floaters.	306 (71.16)
8. Most floaters are benign.	275 (63.95)
9. Some floaters can affect vision, causing visual impairment or even blindness.	260 (60.47)
10. Patients with high myopia and floaters should regularly undergo fundus examinations at the hospital.	340 (79.07)
11. Patients experiencing floaters, whether affecting vision or not, should seek detailed examinations at the hospital.	329 (76.51)
12. Floaters caused by retinal diseases do not require treatment.	295 (68.60)
13. The current examination results for floaters patients being benign does not mean there will never be a problem. If there are sudden flashes, an increase in floaters, or symptoms of obscured vision, a detailed examination is necessary.	329 (76.51)
14. Floaters must always appear in both eyes simultaneously.	290 (67.44)

Regarding attitudes, 57.67% were very much aware that having poor vision in old age is unusual and necessitates a visit to the hospital (A1). Interestingly, 43.72% felt that no treatment was necessary for the time being, even if results were benign after examination (A2), while 61.86% believed that floaters should be treated aggressively (A3). About the possibility of blindness caused by floaters in the future, 54.72% expressed varying degrees of concern (A4). Worryingly, 22.56 and 21.86% reported that floaters had a severe impact on their vision quality (A5) and life and work (A6), respectively (Table 3).

**Table 3:** Responses to attitudes’ dimension items.

Items, *n* (%)	Strongly agree	Agree	Neutral	Disagree	Strongly disagree
1. You believe that it is normal for vision to become unclear as people age, and there is no need to go to the hospital specifically.	22 (5.12)	21 (4.88)	52 (12.09)	87 (20.23)	248 (57.67)
2. You believe that after an examination, if the floaters are benign, there is no need for immediate treatment, and continued observation is sufficient.	77 (17.91)	111 (25.81)	95 (22.09)	53 (12.33)	94 (21.86)
3. You believe that even if floaters are benign at the time of examination, it is important to actively seek treatment; otherwise, the condition will inevitably worsen.	164 (38.14)	102 (23.72)	103 (23.95)	36 (8.37)	25 (5.81)
4. Since experiencing floaters, you often worry that you may eventually lose your vision completely.	118 (27.44)	113 (26.28)	98 (22.79)	55 (12.79)	46 (10.7)
	Very Large	Large	Medium	Small	Very Small
5. Your perception of the impact of floaters on your visual quality.	97 (22.56)	118 (27.44)	126 (29.3)	46 (10.7)	43 (10)
6. Your perception of the impact of floaters on your life and work.	94 (21.86)	107 (24.88)	131 (30.47)	47 (10.93)	51 (11.86)

In terms of practices related to treatment and management, 15.12% of patients had undergone vitreous laser coagulation surgery, 23.26% were planning to do so, while 61.63% expressed no intention to undergo the procedure (P1). Among the 65 who had undergone surgery, 28 reported no postoperative discomfort (P1.1). Additionally, 53.72% would seek immediate medical attention after a visual abnormality (P2). Concerning eye care practices, 44.65% pay close attention to proper eye use (P3), and 36.28% insist on relaxing their eyes every hour (P4). Furthermore, 54.88% have the habit of looking at electronic devices with the lights off, either always or often (P5) (Table 4).

**Table 4:** Responses to practices dimension items.

Items, *n* (%)					
1. Have you received vitreous laser coagulation surgery?					
Yes, I have had laser surgery	65 (15.12)				
Currently no, but planning to receive laser surgery treatment	100 (23.26)				
No, and do not plan to receive laser surgery treatment	265 (61.63)				
	Dry eyes	Eye pain	Sensation of a foreign object in the eye	No discomfort	Not undergone surgery
1.1 If you have undergone vitreous laser coagulation surgery, have you experienced any discomfort symptoms in your eyes?	21 (4.88)	10 (2.33)	6 (1.4)	28 (6.51)	365 (84.88)
	Strongly agree	Agree	Neutral	Disagree	Strongly disagree
2. If I experience an abrupt increase in floaters, see flashes, or encounter obstructions in my vision, I will immediately seek medical attention for a detailed examination.	231 (53.72)	110 (25.58)	71 (16.51)	9 (2.09)	9 (2.09)
3. I practice proper eye usage to avoid eye fatigue.	192 (44.65)	115 (26.74)	91 (21.16)	24 (5.58)	8 (1.86)
4. After 1 h of continuous eye use, I actively allow my eyes to relax and rest.	156 (36.28)	92 (21.4)	119 (27.67)	50 (11.63)	13 (3.02)
	Always	Often	Sometimes	Occasionally	Never
5. How often do you turn off the lights when using electronic devices (e.g., mobile phones, television, and computers)?	134 (31.16)	102 (23.72)	81 (18.84)	42 (9.77)	71 (16.51)

### Correlation analysis among knowledge, attitudes, and practices

Correlation analysis showed a significant positive correlation between knowledge and practices (*r* = 0.239, *p* < 0.001). Regarding the association between attitudes and practices, the analysis revealed an inverse relationship (*r* = −0.219, *p* < 0.001). This suggests that participants reporting more negative attitudes toward floaters were more likely to report negative practices, such as neglecting eye care, while those with more positive attitudes demonstrated more proactive practices (Table 5). The results of the correlation analysis are summarized in Supplementary Table S4. A statistically significant positive correlation was observed between knowledge and practices (*r* = 0.239, *p* < 0.001), indicating that individuals with higher knowledge levels were more likely to engage in proactive practices. Meanwhile, a significant negative correlation was identified between attitudes and practices (*r* = −0.219, *p* < 0.001), suggesting that more negative attitudes were associated with less proactive practices. The correlation between knowledge and attitudes was not statistically significant (*r* = 0.086, *p* = 0.076), highlighting that greater knowledge does not necessarily correspond to more positive attitudes toward floaters.

**Table 5:** Correlation analysis of knowledge, attitudes, and practices.

Variables	Knowledge	Attitudes	Practices
Knowledge	1		
Attitudes	0.086 (*p* = 0.076)	1	
Practices	0.239 (*p* < 0.001)	−0.219 (*p* < 0.001)	1

### Multivariate regression analysis

Multivariate regression analysis demonstrated that patients with a monthly per capita income of 10,000–20,000 yuan (OR = 6.35, 95% CI: [1.734–23.323], *p* = 0.005) and an income of more than 20,000 yuan (OR = 4.402, 95% CI: [1.037–18.686], *p* = 0.045) were more likely to have good knowledge. Furthermore, former (OR = 6.122, 95% CI: [1.431–26.182], *p* = 0.015) or current (OR = 6.270, 95% CI: [1.146–34.305], *p* = 0.034) smokers exhibited better knowledge than those who had never smoked (Supplementary Table S2). Individuals aged 18–40 (OR = 13.060, 95% CI: [1.776–96.052], *p* = 0.012), occasional alcohol consumers (OR = 2.198, 95% CI: [1.139–4.239], *p* = 0.019), and those with more than 1 year since first experiencing floaters were independently associated with positive attitudes (Supplementary Table S3). Higher total knowledge scores (OR = 1.141, 95% CI: [1.069–1.218], *p* < 0.001) were independently associated with proactive practices. However, being myopic with a degree of 300–499 (OR = 0.404, 95% CI: [0.200–0.813], *p* = 0.011), occasional alcohol consumption (OR = 0.499, 95% CI: [0.282–0.885], *p* = 0.017), and 1–2 years since the first experience with floaters (OR = 0.288, 95% CI: [0.106–0.786], *p* = 0.015) were independently associated with proactive practices (Supplementary Table S4).

### SEM analysis

**Figure 1:**
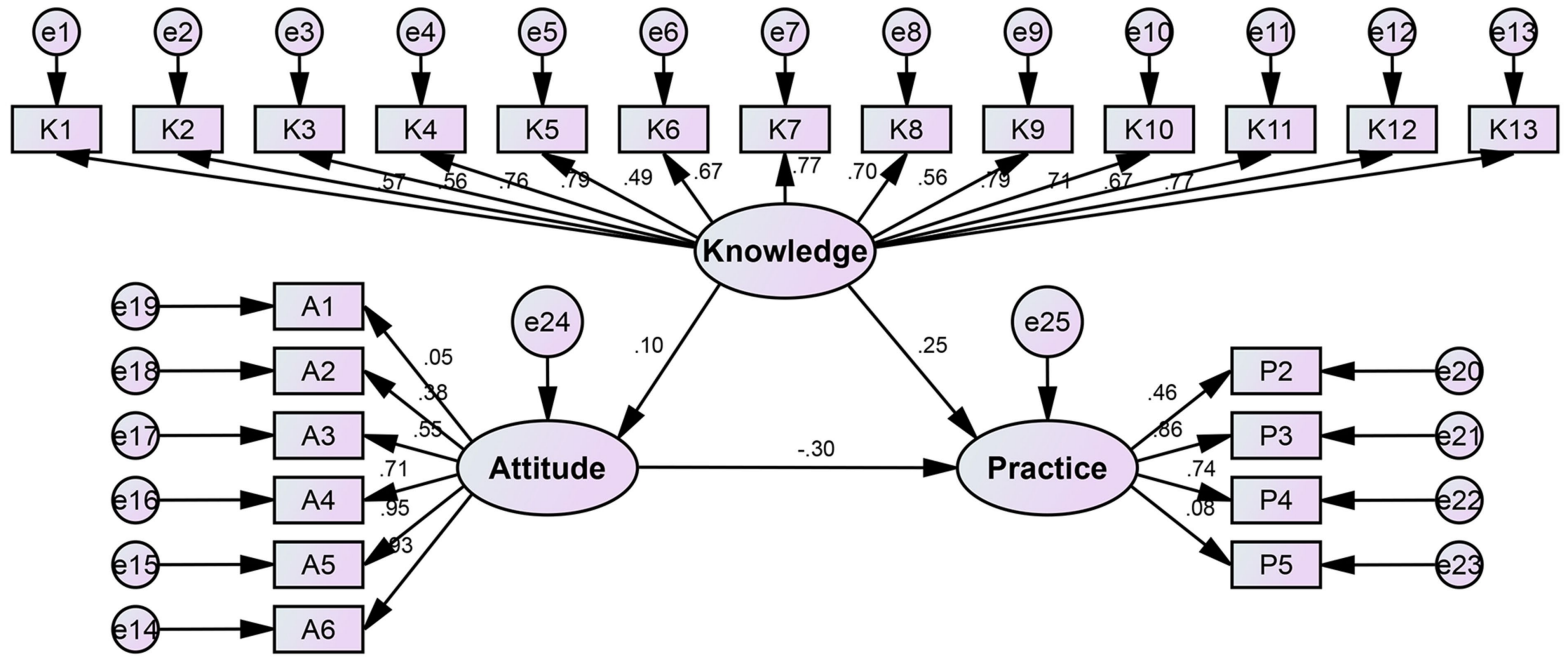
SEM results.

The SEM model showed a good fit (Supplementary Table S5). The SEM analysis revealed that knowledge had a significant direct positive impact on practices (*β* = 0.403, *p* < 0.001), indicating that greater understanding of floaters was associated with more proactive eye care behaviors. In contrast, attitudes exhibited a significant direct negative effect on practices (*β* = −0.112, *p* < 0.001), suggesting that more negative perceptions of floaters may discourage preventive actions. Interestingly, the path from knowledge to attitudes was not statistically significant (*β* = 0.086, *p* = 0.076), implying that increased knowledge alone does not necessarily translate into more positive attitudes. These findings suggest that both cognitive and psychological factors influence patient behavior, and interventions aiming to promote proactive practices should simultaneously address knowledge gaps and negative perceptions (Figure 1; Supplementary Table S6).

## Discussion

Patients exhibited suboptimal knowledge and negative attitudes toward floaters but engaged in some proactive practices, highlighting the need for tailored educational interventions. These interventions should target knowledge gaps and negative attitudes, aiming to facilitate informed decision-making and promote proactive clinical practices.

Our study underscores a critical gap in understanding and managing this ocular phenomenon, indicating the necessity for targeted interventions to enhance patient awareness and foster positive attitudes and behaviors. These results align with existing research emphasizing the pivotal role of patient education in improving health-related knowledge and attitudes (13). It is noteworthy that while the negative correlation between attitudes and practices (*r* = −0.219) was statistically significant, the correlation coefficient indicates a weak to moderate relationship. From a clinical perspective, it suggests that negative attitudes toward floaters may modestly influence patients’ adherence to recommended eye care practices. This finding has practical implications for clinical management, indicating that addressing patients’ negative perceptions about floaters could potentially improve their compliance with preventive eye care practices. However, the moderate strength of this correlation also suggests that other factors beyond attitudes likely influence patients’ eye care behaviors. Clinicians should consider this multifactorial nature when developing patient education and intervention strategies, rather than focusing solely on attitude modification. This approach aligns with holistic patient-centered care models that address both psychological perceptions and practical barriers to optimal healthcare practices (14).

Analyzing the influence of demographic characteristics on KAP scores identified significant variations associated with age, education, employment status, and duration since the first experience of floaters. This aligns with the broader literature, suggesting that sociodemographic factors can impact health-related knowledge and behaviors (15). Notably, the association between monthly per capita income and knowledge scores underscores the role of socioeconomic status in shaping patient understanding, consistent with studies on health disparities (16, 17).

The SEM results elucidate a direct positive impact of knowledge on proactive practices and a negative influence of attitudes on practices. These findings align with previous studies showing that knowledge mediates health-related behaviors (18). The significant positive correlation between knowledge and practices reinforces the importance of informed decision-making in translating knowledge into action (19). Although the correlation coefficient indicates a weak to moderate strength, it holds clinical significance, suggesting that even mildly negative attitudes may hinder patients from adopting beneficial eye care behaviors, such as timely medical consultations or consistent protective habits. Therefore, psychological counseling or targeted communication strategies may be required in addition to knowledge dissemination. In addition to these findings, the SEM results provide further insight into the complex dynamics of knowledge, attitudes, and practices. Specifically, the model revealed a strong direct positive effect of knowledge on practice, suggesting that increased understanding of floaters promotes proactive behaviors such as timely medical visits or eye protection. Conversely, attitudes had a modest but statistically significant negative effect on practice, indicating that concerns or misconceptions may hinder appropriate action. The non-significant path from knowledge to attitudes implies that simply increasing factual knowledge may not be sufficient to alter patients’ beliefs or concerns. This highlights the need for integrated educational and psychological support strategies to effectively improve patient behaviors.

Multivariate analyses identified specific associations, such as higher monthly per capita income and smoking status being linked to better knowledge. These findings align with studies illustrating the impact of income on health knowledge and the potential influence of smoking behaviors on health-related awareness (20, 21). Age, alcohol consumption, and duration since the first experience with floaters were associated with more positive attitudes, consistent with the existing literature on the role of age and health-related attitudes (22, 23).

Higher knowledge scores were independently associated with proactive practices, underscoring the pivotal role of knowledge in shaping patient behavior. Conversely, myopia, occasional alcohol consumption, and a 1–2 year duration since the first experience with floaters were independently associated with less proactive practices. These findings emphasize the importance of targeted interventions for specific subgroups, in line with studies highlighting the impact of lifestyle factors on health-related practices (24, 25).

The response analysis of knowledge-related items revealed a good grasp among participants regarding the primary causes of floaters, such as vitreous degeneration, liquefaction, and detachment. However, misconceptions persist, with a notable portion of respondents inaccurately believing that floaters are contagious and may cause symptoms such as dizziness. These findings highlight areas needing educational interventions, which should clarify the benign nature of most floaters and the importance of professional evaluations for any sudden symptom changes, aligning with previous studies on the impact of patient education in improving health-related knowledge and attitudes (26). Moreover, collaboration with healthcare providers to correct misconceptions could significantly improve patient understanding (27).

Responses regarding attitudes toward floaters show varied beliefs and concerns, particularly around normal vision changes with age and the urgency of treating benign floaters. Many view age-related vision decline as normal and not requiring medical attention, underscoring a need for education on ocular health in older adults. To improve clinical practice, tailored educational interventions should focus on dispelling misconceptions about age-related vision changes, providing evidence-based information on the benign nature of most floaters, and addressing psychological concerns associated with floaters (28). Collaborative efforts between healthcare providers and mental health professionals may enhance the holistic care of individuals with floaters, aligning with studies emphasizing the importance of patient-centered approaches in ophthalmic care (29, 30). Moreover, incorporating psychosocial support and counseling into routine ophthalmic care may contribute to a more comprehensive understanding and management of patients’ attitudes toward floaters (31).

Examining practices associated with floaters, many respondents have not pursued treatments such as vitreous laser coagulation, often due to perceived risks or a lack of information. While most are reluctant to undergo such surgeries, the willingness to seek immediate care for severe symptoms is high. To improve clinical practice, targeted educational interventions should focus on enhancing awareness of the benefits and risks of vitreous laser coagulation surgery, addressing potential concerns or misconceptions that deter individuals from considering this treatment option (2, 32). Additionally, patient education programs should emphasize the importance of regular eye care practices, including proper eye usage, frequent breaks to prevent eye fatigue, and minimizing exposure to artificial light, aligning with studies advocating comprehensive eye health education (33).

This study has several limitations. Its cross-sectional design limited the establishment of causal relationships among KAP. Longitudinal studies would provide a more comprehensive understanding over time. Furthermore, as this study was conducted in a single center in China, the generalizability of our findings to other populations may be limited. Cultural and socioeconomic factors specific to the Chinese context, such as traditional beliefs about eye health, healthcare-seeking behaviors, and access to eye care services, could influence patients’ KAP regarding floaters. The perception and management of floaters might differ substantially in other geographical regions due to variations in healthcare systems, cultural attitudes toward eye symptoms, and economic barriers to accessing eye care. Future multi-center studies across different cultural and socioeconomic settings would be valuable in establishing more globally representative findings. Reliance on self-reported data introduces potential biases, and the study’s geographical specificity may impact generalizability. The use of web-based questionnaires may introduce digital literacy bias, potentially excluding individuals with limited access to digital technology or those uncomfortable with online surveys, particularly among older age groups. Furthermore, the study did not consider hereditary factors as a variable, which could have provided deeper insights into the KAP toward self-management among participants with floaters. Additionally, our analysis did not differentiate between patients with unilateral and bilateral floaters. Recognizing and analyzing these groups separately could yield more tailored insights, given that their experiences and clinical outcomes may significantly vary. Additionally, due to our web-based questionnaire distribution method, we were unable to determine the total number of individuals who viewed the survey link but chose not to participate. As a result, a traditional response rate could not be accurately calculated. This limitation is inherent in the QR code-based distribution approach we used. The absence of response rate data may impact our understanding of potential selection bias, as the characteristics of non-respondents remain unknown.

In conclusion, this study identifies significant deficiencies in patients’ knowledge, attitudes, and practices regarding floaters. To enhance clinical practices, targeted educational initiatives are crucial for addressing knowledge gaps, mitigating negative attitudes, and fostering proactive management. Tailoring interventions for specific demographic groups identified in the analysis will contribute to more effective patient-centered care.

## Data Availability

The original contributions presented in the study are included in the article/Supplementary material, further inquiries can be directed to the corresponding author.

## Supplementary material

SUPPLEMENTARY TABLE S1 CFA results.

SUPPLEMENTARY TABLE S2 Univariate and multivariate regression analysis of knowledge.

SUPPLEMENTARY TABLE S3 Univariate and multivariate regression analysis of attitudes.

SUPPLEMENTARY TABLE S4 Univariate and multivariate regression analysis of practices.

SUPPLEMENTARY TABLE S5 SEM model fit indicators.

SUPPLEMENTARY TABLE S6 SEM results.

## References

[1] Fan WS Huang SY Nguyen HT Ho WT Chao WH Lin FC. Design of a Functional eye Dressing for treatment of the vitreous floater. J Pers Med. (2022) 12:1659. doi: 10.3390/jpm12101659, PMID: 36294798 PMC9604789

[2] Souza CE Lima LH Nascimento H Zett C Belfort R Jr. Objective assessment of Yag laser Vitreolysis in patients with symptomatic vitreous floaters. Int J Retina Vitreous. (2020) 6:1. doi: 10.1186/s40942-019-0205-8, PMID: 31988795 PMC6971902

[3] Ma JW Hung JL Takeuchi M Shieh PC Horng CT. A new pharmacological Vitreolysis through the supplement of mixed fruit enzymes for patients with ocular floaters or vitreous hemorrhage-induced floaters. J Clin Med. (2022) 11:6710. doi: 10.3390/jcm11226710, PMID: 36431188 PMC9695351

[4] Webb BF Webb JR Schroeder MC North CS. Prevalence of vitreous floaters in a community sample of smartphone users. Int J Ophthalmol. (2013) 6:402–5. doi: 10.3980/j.issn.2222-3959.2013.03.27, PMID: 23826541 PMC3693028

[5] Liu X Wang Q Zhao J. Acute retinal detachment after Nd: Yag treatment for vitreous floaters and Postertior capsule opacification: a case report. BMC Ophthalmol. (2020) 20:157. doi: 10.1186/s12886-020-01428-7, PMID: 32306922 PMC7168856

[6] Wu RH Zhang R Lin Z Liang QH Moonasar N. A comparison between topical and Retrobulbar anesthesia in 27-gauge vitrectomy for vitreous floaters: a randomized controlled trial. BMC Ophthalmol. (2018) 18:164. doi: 10.1186/s12886-018-0838-7, PMID: 29981573 PMC6035792

[7] Kwak C Seo YJ Park KH Han W. Analysis of the knowledge, attitudes, and practice model of healthcare professionals on hearing loss at elderly dementia residences in Korea. Healthcare. (2022) 10:792. doi: 10.3390/healthcare10050792, PMID: 35627929 PMC9140935

[8] Lu Y Liu C Fawkes S Wang Z Yu D. Knowledge, attitudes, and practice of general practitioners toward community detection and Management of Mild Cognitive Impairment: a cross-sectional study in Shanghai, China. BMC Prim Care. (2022) 23:114. doi: 10.1186/s12875-022-01716-9, PMID: 35545764 PMC9092880

[9] Katsanos A Tsaldari N Gorgoli K Lalos F Stefaniotou M Asproudis I. Safety and efficacy of Yag laser Vitreolysis for the treatment of vitreous floaters: an overview. Adv Ther. (2020) 37:1319–27. doi: 10.1007/s12325-020-01261-w, PMID: 32086749 PMC7140748

[10] Lumi X Hawlina M Glavač D Facskó A Moe MC Kaarniranta K. Ageing of the vitreous: from acute onset floaters and flashes to retinal detachment. Ageing Res Rev. (2015) 21:71–7. doi: 10.1016/j.arr.2015.03.006, PMID: 25841656

[11] Milston R Madigan MC Sebag J. Vitreous floaters: etiology, diagnostics, and management. Surv Ophthalmol. (2016) 61:211–27. doi: 10.1016/j.survophthal.2015.11.008, PMID: 26679984

[12] Lee F Suryohusodo AA. Knowledge, attitude, and practice assessment toward Covid-19 among communities in East Nusa Tenggara, Indonesia: a cross-sectional study. Front Public Health. (2022) 10:957630. doi: 10.3389/fpubh.2022.957630, PMID: 36388283 PMC9659730

[13] Feng YY Chaves GSS Shi W Pakosh M Zhang L Gallagher R. Education interventions in Chinese cardiac patients on health Behaviours, disease-related knowledge, and health outcomes: a systematic review and Meta-analysis. Patient Educ Couns. (2021) 104:1018–29. doi: 10.1016/j.pec.2020.12.001, PMID: 33349505

[14] Street RL Jr Makoul G Arora NK Epstein RM. How does communication heal? Pathways linking clinician-patient communication to health outcomes. Patient Educ Couns. (2009) 74:295–301. doi: 10.1016/j.pec.2008.11.015, PMID: 19150199

[15] Mikkelsen HT Småstuen MC Haraldstad K Helseth S Skarstein S Rohde G. Changes in health-related quality of life in adolescents and the impact of gender and selected variables: a two-year longitudinal study. Health Qual Life Outcomes. (2022) 20:123. doi: 10.1186/s12955-022-02035-4, PMID: 35982467 PMC9387404

[16] Carrera PM Calderazzo S. Knowledge of Cancer risk factors and risk-reduction in high-income countries. Prev Med. (2023) 173:107583. doi: 10.1016/j.ypmed.2023.107583, PMID: 37352940

[17] Santos JA McKenzie B Rosewarne E Hogendorf M Trieu K Woodward M. Strengthening knowledge to practice on effective salt reduction interventions in low-and middle-income countries. Curr Nutr Rep. (2021) 10:211–25. doi: 10.1007/s13668-021-00365-1, PMID: 34224108

[18] Liu P Teng M Han C. How does environmental knowledge translate into pro-environmental behaviors?: the mediating role of environmental attitudes and behavioral intentions. Sci Total Environ. (2020) 728:138126. doi: 10.1016/j.scitotenv.2020.138126, PMID: 32361356

[19] Stolwijk ML van Nispen RMA van der Ham AJ Veenman E van Rens G. Barriers and facilitators in the referral pathways to low vision services from the perspective of patients and professionals: a qualitative study. BMC Health Serv Res. (2023) 23:64. doi: 10.1186/s12913-022-09003-0, PMID: 36681848 PMC9860223

[20] Marsh EE Al-Hendy A Kappus D Galitsky A Stewart EA Kerolous M. Burden, prevalence, and treatment of uterine fibroids: a survey of U.S. women. J Womens Health. (2018) 27:1359–67. doi: 10.1089/jwh.2018.7076, PMID: 30230950 PMC6247381

[21] Park J Lim MK Yun EH Oh JK Jeong BY Cheon Y. Influences of tobacco-related knowledge on awareness and behavior towards smoking. J Korean Med Sci. (2018) 33:e302. doi: 10.3346/jkms.2018.33.e302, PMID: 30450026 PMC6236079

[22] Brannon D Miller CJ. What's my age again? The influence of subjective age on consumer health-related attitudes. Health Mark Q. (2019) 36:254–70. doi: 10.1080/07359683.2019.1618011, PMID: 31169083

[23] Deeks A Lombard C Michelmore J Teede H. The effects of gender and age on health related behaviors. BMC Public Health. (2009) 9:213. doi: 10.1186/1471-2458-9-213, PMID: 19563685 PMC2713232

[24] Dimitrov Ulian M Pinto AJ de Morais Sato P Benatti FB Lopes de Campos-Ferraz P Coelho D. Effects of a new intervention based on the Health at Every Size approach for the management of obesity: The "Health and Wellness in Obesity" study. PLoS One. (2018) 13:e0198401. doi: 10.1371/journal.pone.0198401, PMID: 29979699 PMC6034785

[25] Kumar S Badiyani BK Lalani A Kumar A Roy S. Influence of lifestyle factors on Oral health-related quality of life in pregnant women in Indore City. Malays J Med Sci. (2018) 25:126–32. doi: 10.21315/mjms2018.25.2.13, PMID: 30918462 PMC6422588

[26] Pueyo-Garrigues M Whitehead D Pardavila-Belio MI Canga-Armayor A Pueyo-Garrigues S Canga-Armayor N. Health education: a Rogerian concept analysis. Int J Nurs Stud. (2019) 94:131–8. doi: 10.1016/j.ijnurstu.2019.03.005, PMID: 30951988

[27] Chlasta-Twardzik E Nowińska A Wylęgała E. Acute macular edema and serous detachment on the first day after phacoemulsification surgery: a case report. Am J Ophthalmol Case Rep. (2020) 20:100905. doi: 10.1016/j.ajoc.2020.100905, PMID: 32954045 PMC7486609

[28] Nejat F Jadidi K Aghamollaei H Nejat MA Nabavi NS Eghtedari S. The assessment of the concentration of candidate cytokines in response to conjunctival-exposure of atmospheric low-temperature plasma in an animal model. BMC Ophthalmol. (2021) 21:417. doi: 10.1186/s12886-021-02167-z, PMID: 34863132 PMC8642870

[29] Ehrlich JR Spaeth GL Carlozzi NE Lee PP. Patient-centered outcome measures to assess functioning in randomized controlled trials of low-vision rehabilitation: a review. Patient. (2017) 10:39–49. doi: 10.1007/s40271-016-0189-5, PMID: 27495171

[30] Talley-Rostov A. Patient-centered care and refractive cataract surgery. Curr Opin Ophthalmol. (2008) 19:5–9. doi: 10.1097/ICU.0b013e3282f2d7a3, PMID: 18090889

[31] Lieb M Tagay S Breidenstein A Hepp T Le Guin CHD Scheel J. Psychosocial impact of prognostic genetic testing in uveal melanoma patients: a controlled prospective clinical observational study. BMC Psychol. (2020) 8:8. doi: 10.1186/s40359-020-0371-3, PMID: 32005293 PMC6995105

[32] Shrestha A Khatri B Naito T. A unique experience of retinal diseases screening in Nepal. Clin Ophthalmol. (2020) 14:2037–42. doi: 10.2147/opth.S259274, PMID: 32764869 PMC7382583

[33] Javitt JC. Preventing blindness in Americans: the need for eye health education. Surv Ophthalmol. (1995) 40:41–4. doi: 10.1016/s0039-6257(95)80045-x, PMID: 8545801

