# Supplementary material for: Knowledge, attitudes, and practices regarding floaters among patients
Source: Front Med (Lausanne). 2025 Jul 9;12:1579435. doi: 10.3389/fmed.2025.1579435 (PMC12283980; doi:10.3389/fmed.2025.1579435)
Supplement: SUPPLEMENTARY TABLE S1 — CFA results. [file Table_1.docx]

**Table S1. CFA Results**

|  |  |  | **Estimate** | **Standardized Estimate** | **S.E.** | **C.R.** | **P** |
| --- | --- | --- | --- | --- | --- | --- | --- |
| K1 | <--- | Knowledge | 1.000 | 0.572 |  |  |  |
| K2 | <--- | Knowledge | 1.023 | 0.562 | 0.106 | 9.694 | <0.001 |
| K3 | <--- | Knowledge | 1.207 | 0.762 | 0.101 | 11.925 | <0.001 |
| K4 | <--- | Knowledge | 1.233 | 0.792 | 0.101 | 12.200 | <0.001 |
| K5 | <--- | Knowledge | 0.899 | 0.487 | 0.104 | 8.672 | <0.001 |
| K6 | <--- | Knowledge | 1.222 | 0.674 | 0.111 | 11.016 | <0.001 |
| K7 | <--- | Knowledge | 1.288 | 0.769 | 0.107 | 11.984 | <0.001 |
| K8 | <--- | Knowledge | 1.248 | 0.703 | 0.110 | 11.329 | <0.001 |
| K9 | <--- | Knowledge | 1.006 | 0.556 | 0.105 | 9.616 | <0.001 |
| K10 | <--- | Knowledge | 1.195 | 0.794 | 0.098 | 12.222 | <0.001 |
| K11 | <--- | Knowledge | 1.111 | 0.709 | 0.098 | 11.392 | <0.001 |
| K12 | <--- | Knowledge | 1.144 | 0.666 | 0.105 | 10.936 | <0.001 |
| K13 | <--- | Knowledge | 1.214 | 0.774 | 0.101 | 12.039 | <0.001 |
| A1 | <--- | Attitude | 1.000 | 0.055 |  |  |  |
| A2 | <--- | Attitude | 8.604 | 0.385 | 7.844 | 1.097 | 0.273 |
| A3 | <--- | Attitude | 10.427 | 0.546 | 9.459 | 1.102 | 0.270 |
| A4 | <--- | Attitude | 14.669 | 0.707 | 13.280 | 1.105 | 0.269 |
| A5 | <--- | Attitude | 18.626 | 0.952 | 16.840 | 1.106 | 0.269 |
| A6 | <--- | Attitude | 18.666 | 0.929 | 16.877 | 1.106 | 0.269 |
| P2 | <--- | Practice | 1.000 | 0.463 |  |  |  |
| P3 | <--- | Practice | 2.004 | 0.863 | 0.245 | 8.170 | <0.001 |
| P4 | <--- | Practice | 1.931 | 0.739 | 0.226 | 8.540 | <0.001 |
| P5 | <--- | Practice | 0.264 | 0.081 | 0.175 | 1.505 | 0.132 |
