# Supplementary material for: Knowledge, attitudes, and practices regarding floaters among patients
Source: Front Med (Lausanne). 2025 Jul 9;12:1579435. doi: 10.3389/fmed.2025.1579435 (PMC12283980; doi:10.3389/fmed.2025.1579435)
Supplement: SUPPLEMENTARY TABLE S2 — Univariate and multivariate regression analysis of knowledge. [file Table_2.docx]

**Table S2. Responses to Knowledge Dimension Items**

| **Items, n (%)** | **Correct Rate** |
| --- | --- |
| **1. Floaters are generally caused by vitreous degeneration, which is an aging phenomenon.** | 285 (66.28) |
| **2. Vitreous liquefaction and posterior vitreous detachment are the main causes of floaters.** | 254 (59.07) |
| **3. Floaters can be transmitted to people around you.** | 326 (75.81) |
| **4. Floaters may be limited to one eye or may occur in both eyes.** | 331 (76.98) |
| **5. Floaters can cause discomfort symptoms such as dizziness.** | 203 (47.21) |
| **6. Floaters often occur in middle-aged and elderly people over 40, highly myopic individuals, and those who have had cataract surgery.** | 257 (59.77) |
| **7. Conditions such as high blood pressure, diabetes, intraocular inflammation, retinal holes, and trauma can also lead to floaters.** | 306 (71.16) |
| **8. Most floaters are benign.** | 275 (63.95) |
| **9. Some floaters can affect vision, causing visual impairment or even blindness.** | 260 (60.47) |
| **10. Patients with high myopia and floaters should regularly undergo fundus examinations at the hospital.** | 340 (79.07) |
| **11. Patients experiencing floaters, whether affecting vision or not, should seek detailed examinations at the hospital.** | 329 (76.51) |
| **12. Floaters caused by retinal diseases do not require treatment.** | 295 (68.60) |
| **13. The current examination results for floaters patients being benign does not mean there will never be a problem. If there are sudden flashes, an increase in floaters, or symptoms of obscured vision, a detailed examination is necessary.** | 329 (76.51) |
| **14. Floaters must always appear in both eyes simultaneously.** | 290 (67.44) |
