# Supplementary material for: Knowledge, attitudes, and practices regarding floaters among patients
Source: Front Med (Lausanne). 2025 Jul 9;12:1579435. doi: 10.3389/fmed.2025.1579435 (PMC12283980; doi:10.3389/fmed.2025.1579435)
Supplement: SUPPLEMENTARY TABLE S3 — Univariate and multivariate regression analysis of attitudes. [file Table_3.docx]

**Table S3. Responses to Attitudes Dimension Items**

| **Items, n (%)** | **Strongly Agree** | **Agree** | **Neutral** | **Disagree** | **Strongly Disagree** |
| --- | --- | --- | --- | --- | --- |
| **1. You believe that it is normal for vision to become unclear as people age, and there is no need to go to the hospital specifically.** | 22 (5.12) | 21 (4.88) | 52 (12.09) | 87 (20.23) | 248 (57.67) |
| **2. You believe that after an examination, if floaters are benign, there is no need for immediate treatment, and continued observation is sufficient.** | 77 (17.91) | 111 (25.81) | 95 (22.09) | 53 (12.33) | 94 (21.86) |
| **3. You believe that even if floaters are benign at the time of examination, it is important to actively seek treatment; otherwise, the condition will inevitably worsen.** | 164 (38.14) | 102 (23.72) | 103 (23.95) | 36 (8.37) | 25 (5.81) |
| **4. Since experiencing floaters, you often worry that you may eventually lose your vision completely.** | 118 (27.44) | 113 (26.28) | 98 (22.79) | 55 (12.79) | 46 (10.7) |
|  | **Very Large** | **Large** | **Medium** | **Small** | **Very Small** |
| **5. Your perception of the impact of floaters on your visual quality.** | 97 (22.56) | 118 (27.44) | 126 (29.3) | 46 (10.7) | 43 (10) |
| **6. Your perception of the impact of floaters on your life and work.** | 94 (21.86) | 107 (24.88) | 131 (30.47) | 47 (10.93) | 51 (11.86) |
