# Supplementary material for: Knowledge, attitudes, and practices regarding floaters among patients
Source: Front Med (Lausanne). 2025 Jul 9;12:1579435. doi: 10.3389/fmed.2025.1579435 (PMC12283980; doi:10.3389/fmed.2025.1579435)
Supplement: SUPPLEMENTARY TABLE S4 — Univariate and multivariate regression analysis of practices. [file Table_4.docx]

**Table S4. Responses to Practices Dimension Items**

| **1. Have you received vitreous laser coagulation surgery?** |  |  |  |  |  |
| --- | --- | --- | --- | --- | --- |
| **Yes, I have had laser surgery** | 65 (15.12) |  |  |  |  |
| **Currently no, but planning to receive laser surgery treatment** | 100 (23.26) |  |  |  |  |
| **No, and do not plan to receive laser surgery treatment** | 265 (61.63) |  |  |  |  |
|  | **Dry eyes** | **Eye pain** | **Sensation of foreign object in the eye** | **No discomfort** | **Not undergone surgery** |
| **1.1 If you have undergone vitreous laser coagulation surgery, have you experienced any discomfort symptoms in your eyes?** | 21 (4.88) | 10 (2.33) | 6 (1.4) | 28 (6.51) | 365 (84.88) |
|  | **Strongly Agree** | **Agree** | **Neutral** | **Disagree** | **Strongly Disagree** |
| **2.** **If I experience an abrupt increase in floaters, see flashes, or encounter obstructions in my vision, I will immediately seek medical attention for a detailed examination.** | 231 (53.72) | 110 (25.58) | 71 (16.51) | 9 (2.09) | 9 (2.09) |
| **3.** **I practice proper eye usage to avoid eye fatigue.** | 192 (44.65) | 115 (26.74) | 91 (21.16) | 24 (5.58) | 8 (1.86) |
| **4.** **After one hour of continuous eye use, I actively allow my eyes to relax and rest.** | 156 (36.28) | 92 (21.4) | 119 (27.67) | 50 (11.63) | 13 (3.02) |
|  | **Always** | **Often** | **Sometimes** | **Occasionally** | **Never** |
| **5. How often do you turn off the lights when using electronic devices (e.g., mobile phones, television, computers, etc.)?** | 134 (31.16) | 102 (23.72) | 81 (18.84) | 42 (9.77) | 71 (16.51) |
