# Supplementary material for: Knowledge, attitudes, and practices regarding floaters among patients
Source: Front Med (Lausanne). 2025 Jul 9;12:1579435. doi: 10.3389/fmed.2025.1579435 (PMC12283980; doi:10.3389/fmed.2025.1579435)
Supplement: SUPPLEMENTARY TABLE S5 — SEM model fit indicators. [file Table_5.docx]

**Table S5. Correlation Analysis of Knowledge, Attitudes, and Practices**

|  | **Knowledge** | **Attitude** | **Practice** |
| --- | --- | --- | --- |
| Knowledge | 1 |  |  |
| Attitude | 0.086 (P=0.076) | 1 |  |
| Practice | 0.239 (P<0.001) | -0.219 (P<0.001) | 1 |
