# Supplementary material for: Knowledge, attitudes, and practices regarding floaters among patients
Source: Front Med (Lausanne). 2025 Jul 9;12:1579435. doi: 10.3389/fmed.2025.1579435 (PMC12283980; doi:10.3389/fmed.2025.1579435)
Supplement: SUPPLEMENTARY TABLE S6 — SEM results. [file Table_6.docx]

**Table S6. SEM Results**

|  |  |  | **Estimate** | **S.E.** | **C.R.** | **P** |
| --- | --- | --- | --- | --- | --- | --- |
| Attitude | <--- | Knowledge | .438 | .225 | 1.952 | 0.051 |
| Practice | <--- | Knowledge | .403 | .100 | 4.019 | <0.001 |
| Practice | <--- | Attitude | -.112 | .023 | -4.844 | <0.001 |
